# Prognostic Implications of Thrombocytopenia in Chinese Patients With Newly Diagnosed Multiple Myeloma

**DOI:** 10.1002/cam4.71353

**Published:** 2025-11-02

**Authors:** Xiaojing Li, Xiaohui Lai, Xiaolin Wang, Qiang Liu, Xin Liu, Luqun Wang, Jun Peng, Ping Chen, Hai Zhou

**Affiliations:** ^1^ Department of Hematology Qilu Hospital of Shandong University Jinan China; ^2^ Fujian Institute of Hematology, Fujian Provincial Key Laboratory on Hematology Fujian Medical University Union Hospital Fuzhou China; ^3^ Department of Hematology Shandong Provincial Hospital Affiliated to Shandong First Medical University Jinan China; ^4^ Shandong Provincial Key Laboratory of Immunohematology, Qilu Hospital of Shandong University Jinan China; ^5^ Shandong Provincial Clinical Medicine Research Center for Hematology, Qilu Hospital of Shandong University Jinan China

**Keywords:** multiple myeloma, prognosis, thrombocytopenia

## Abstract

**Background:**

Thrombocytopenia is less common but shows high risk of early mortality in newly diagnosed multiple myeloma (NDMM) patients. In the era of novel agents‐based induction therapy (NAIT), it is unclear whether NAIT can overcome the poor prognosis associated with thrombocytopenia.

**Objectives:**

To evaluate the prognostic implications of thrombocytopenia in NDMM patients.

**Methods:**

We retrospectively analyzed 1363 NDMM patients baseline characteristics, treatment response and survival, further performed regression analysis, constructed a nomogram model to predict progression free survival (PFS), and further internally validated this model.

**Results:**

Overall, 211 (15.48%) NDMM patients were harboring thrombocytopenia, with advanced disease stages and worse outcomes. Their PFS (15 months vs 21.5 months, *p* < 0.001)and overall survival (47 months vs 77 months, *p* < 0.001) were significantly inferior compared with patients without thrombocytopenia. In NDMM receiving NAIT, the overall response (87.8% vs 92.4%, *p* = 0.33) but not deep response or survival could be improved between patients with and without thrombocytopenia. Five important variables (thrombocytopenia, R‐ISS stage III, NAIT, deep response and autologous stem‐cell transplantation) in multivariate Cox analysis were incorporated in the nomogram, which was further validated by internal datasets. The Calibration curve and time‐dependent Receiver operating characteristic showed that the model accurately predicted the 12‐ and 24‐ months PFS of NDMM patients.

**Conclusions:**

Thrombocytopenia has an indispensable prognostic effect in decreasing responses to induction therapy and survival in NDMM patients. Thrombocytopenia might need to be regarded as an independent prognostic factor in risk stratification of Chinese NDMM patients.

## 1 Introduction

Multiple myeloma (MM) is the second most common hematological malignancy, characterized by abnormal plasma cell proliferation and, consequently, leading to end‐organ damage [1, 2, 3, 4]. Anemia and thrombocytopenia can be seen in a subset of newly diagnosed MM (NDMM) patients [5]. Thrombocytopenia is less common but shows a high risk of early mortality [6]. In the development of the International Staging System (ISS) for MM in 2005, platelet count was applied in the preliminary prognostic factor analysis, univariate and multivariate survival analysis, and was found to be a powerful predictor of survival [7]. However, platelet count was not incorporated into ISS, and the prognostic value of thrombocytopenia in NDMM patients might have been underestimated. After nearly two decades with a great revolution in myeloma treatment and improvement of survival, Mao et al. developed an individualized and weighted myeloma prognostic score system (MPSS) in NDMM patients [8]. Thrombocytopenia was integrated into MPSS and assigned a point equal to that of ISS stage III and two or more high‐risk cytogenetic abnormalities (HRCA). Recent studies presented similar results that thrombocytopenia at diagnosis was linked to poor prognosis in MM patients [9, 10]. These previous reports highlighted the importance of thrombocytopenia in the risk stratification of NDMM patients.

Current international guidelines favor triplet or quadruplet induction regimens consisting of proteasome inhibitors, immunomodulatory agents, and monoclonal antibodies. Adequate induction therapy greatly improves the prognosis and survival of NDMM patients [11, 12]. Proteasome inhibitors combined with lenalidomide and dexamethasone are strongly recommended as the standard frontline induction regimens based on the superior progression‐free survival (PFS) and overall survival (OS) in the previously reported blockbuster studies in NDMM patients [13, 14, 15, 16, 17, 18, 19]. Recent studies have focused on whether the addition of monoclonal antibodies to triplet induction regimens can further improve the efficacy. Overall, the quadruplet regimens achieved better outcomes than the triplet regimens [20, 21, 22, 23]. However, it is unclear whether triplet or quadruplet induction regimens are sufficient for NDMM patients with thrombocytopenia.

This study aimed to evaluate the prognostic implications of thrombocytopenia in NDMM patients. We analyzed the baseline clinical features, responses to frontline induction therapy, and survival between NDMM patients with and without thrombocytopenia. Based on the results of Cox regression analysis, we constructed a nomogram to predict 12‐ and 24‐month PFS, and further validated this model.

## 2 Methods

### 2.1 Study Design and Participants

We conducted a retrospective, multi‐center study and enrolled 1363 NDMM patients who received induction therapies from three hospitals in China between January 2015 and December 2023. Diagnosis was in accordance with the International Myeloma Working Group (IMWG) criteria [24]. Patients diagnosed as primary amyloidosis (PAL), plasma cell leukemia (PCL), monoclonal gammopathy of undetermined significance (MGUS), and smoldering multiple myeloma (SMM) were excluded. The primary endpoint was PFS, and the secondary endpoints were responses and OS. The study was approved by the Ethical Committee of Qilu Hospital of Shandong University and conducted in accordance with the Declaration of Helsinki. Informed consents were obtained from patients before recruitment.

Thrombocytopenia was defined as an absolute platelet count less than 100,000/uL in peripheral blood in NDMM patients. Novel agents accessible to Chinese MM patients included proteasome inhibitors (bortezomib, ixazomib, and carfilzomib), immunomodulatory agents (thalidomide, lenalidomide, and pomalidomide), and anti‐CD38 monoclonal antibodies (daratumumab). In this study, novel agent‐based induction therapy (NAIT) was defined as a triplet or quadruplet induction regimen consisting of at least two novel agents and accompanying steroids.

IMWG consensus criteria for response assessment were used to evaluate the response and progression [25]. Patients were categorized as having stringent complete response (sCR), complete response (CR), very good partial response (VGPR), partial response (PR), stable disease (SD), and progressive disease (PD).

PFS was defined as the duration from diagnosis to disease progression, first relapse, death, or the end of follow‐up, whichever comes first. OS was defined as the duration from diagnosis to death or the end of follow‐up. Moreover, if patients' outcomes were not present at the end of follow‐up, such case information was defined as censored data [26].

### 2.2 Statistical Analysis

Baseline clinical characteristics of NDMM patients with low and normal platelet count were compared using the Chi‐square test and Fisher's exact test. Probabilities for PFS and OS were estimated using the Kaplan–Meier curve, and differences were tested for statistical significance using the two‐sided log‐rank test. We used univariate logistic regression analysis to investigate the impact of induction regimens and platelet count on efficacy, as well as univariate Cox regression analysis to evaluate the effects of variables on PFS and OS. Moreover, variables with statistical significance in the univariate Cox regression analysis and meeting the proportionality assumption were included in the subsequent multivariate Cox regression analysis. Missing data were considered as dummy variables.

The dataset was interpolated to form a complete dataset by random forest interpolation and later divided into the training and validation sets in a ratio of 7:3. Based on the results of multivariate Cox regression analysis, we constructed a nomogram for PFS in the complete dataset. Calibration plots and time‐dependent Receiver Operating Characteristic (ROC) curves were used to evaluate the predictive accuracy and conformity in training and validation datasets.

All statistical tests were two‐sided, and *p* values less than 0.05 were considered significant. All statistical analyses were performed in SPSS software version 26.0 (IBBM Corp). The Kaplan–Meier survival curves, the nomogram, time‐dependent ROC curve, and the calibration curves were constructed in R software version 4.3.3 (R Project for Statistical Computing, Vienna, Austria).

## 3 Results

### 3.1 Patient Characteristics

**FIGURE 1:**
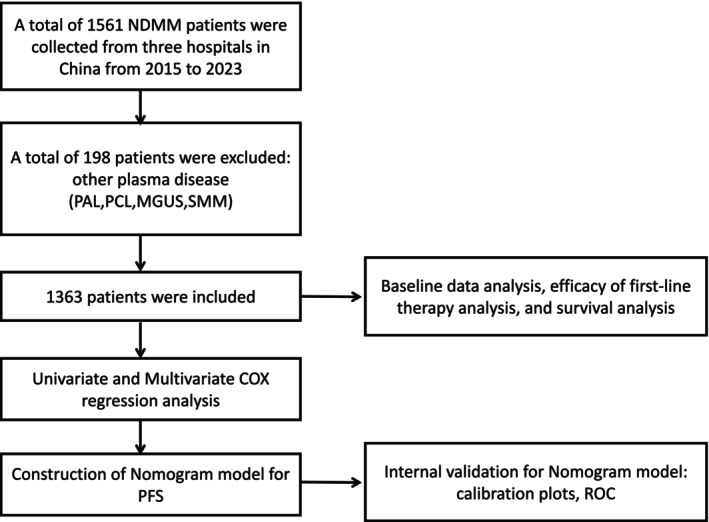
The flow diagram of the study. Abbreviations: MGUS, monoclonal gammopathy of undetermined significance; NDMM, newly diagnosed multiple myeloma; PAL, primary amyloidosis; PCL, plasma cell leukemia; PFS, progression‐free survival; ROC, Receiver operating characteristic analysis; SMM, smoldering multiple myeloma.

As shown in Figure 1, 1363 NDMM patients were analyzed. The incidence of thrombocytopenia in NDMM patients was 15.48% (211/1363, Table 1). The gerontal NDMM patients with thrombocytopenia were more than those with a normal platelet count, especially those over 70 years (20.4% vs 11.8%, *p* = 0.003). NDMM patients with thrombocytopenia presented a larger number of bone marrow plasma cells (*p* < 0.001), more advanced Durie‐Salmon (DS) stage (more severe anemia and hypercalcemia, *p* < 0.001), ISS stage [lower albumin and higher β2‐microglobulin (β2‐MG) levels, *p* < 0.001] and revised ISS (R‐ISS) stage [higher lactate dehydrogenase (LDH) levels and more patients with HRCA, *p* < 0.001] than patients with a normal platelet count (Table 1). NDMM patients with and without thrombocytopenia received comparable induction regimens. The most commonly used induction therapy was bortezomib, lenalidomide, and dexamethasone (VRD); the second most commonly used therapy was bortezomib, thalidomide, and dexamethasone (VTD); and then the triplet therapy consisting of daratumumab and proteasome inhibitors. After induction therapy, patients with a normal platelet count receiving autologous stem cell transplantation (ASCT) were a little more than patients with thrombocytopenia (25.3% vs 18.5%, *p* = 0.033). Interestingly, the ratio of males to females in NDMM patients with thrombocytopenia was 1.78, while this ratio was 1.16 in patients with a normal platelet count.

**TABLE 1:** Characteristics of 1363 patients with newly diagnosed multiple myeloma.

Patients' characteristics	All patients (*N* = 1363)	Patients without thrombocytopenia (*N* = 1152)	Patients with thrombocytopenia (*N* = 211)	*p*
Sex				0.006
Female	610	534 (46.4%)	76 (36.0%)	
Male	753	618 (53.6%)	135 (64.0%)	
Age (Years)				0.003
< 60	668	577 (50.1%)	91 (43.1%)	
60–70	516	439 (38.1%)	77 (36.5%)	
> 70	179	136 (11.8%)	43 (20.4%)	
BMPCs (%)				< 0.001
< 60	1147	995 (86.4%)	152 (72.0%)	
≥ 60	182	131 (11.4%)	51 (24.2%)	
Missing	34	26 (2.3%)	8 (3.8%)	
Serum calcium (mmol/L)				0.183
< 2.65	1200	1020 (88.5%)	180 (85.3%)	
≥ 2.65	163	132 (11.5%)	31 (14.7%)	
Serum creatinine (umol/L)				< 0.001
< 177	1125	970 (84.2%)	155 (73.5%)	
≥ 177	238	182 (15.8%)	56 (26.5%)	
Hb (g/L)				< 0.001
≥ 85	857	803 (69.7%)	54 (25.6%)	
< 85	506	349 (30.3%)	157 (74.4%)	
Bone destruction				0.704
< 3 sites	686	578 (50.2%)	108 (51.2%)	
≥ 3 sites	570	486 (42.2%)	84 (39.8%)	
Missing	107	88 (7.6%)	19 (9.0%)	
DS stage				< 0.001
I	94	92 (8.0%)	2 (0.9%)	
II	338	315 (27.3%)	23 (10.9%)	
III	907	721 (62.6%)	186 (88.2%)	
Missing	24	24 (2.1%)	0 (0.0%)	
β2‐MG (mg/L)				< 0.001
< 5.5	784	716 (62.2%)	68 (32.2%)	
≥ 5.5	575	433 (37.6%)	142 (67.3%)	
Missing	4	3 (0.3%)	1 (0.5%)	
Albumin (g/L)				< 0.001
≥ 35	804	710 (61.6%)	94 (44.5%)	
< 35	559	442 (38.4%)	117 (55.5%)	
ISS stage				< 0.001
I	356	337 (29.3%)	19 (9.0%)	
II	428	379 (32.9%)	49 (23.2%)	
III	575	433 (37.6%)	142 (67.3%)	
Missing	4	3 (0.3%)	1 (0.5%)	
LDH				< 0.001
Normal	1047	911 (79.1%)	136 (64.5%)	
Elevated^a^	316	241 (20.9%)	75 (35.5%)	
HRCA^b^				< 0.001
No	406	364 (31.6%)	42 (19.9%)	
Yes	503	403 (35.0%)	100 (47.4%)	
Missing	454	385 (33.4%)	69 (32.7%)	
R‐ISS stage				< 0.001
I	160	155 (13.5%)	5 (2.4%)	
II	697	615 (53.4%)	82 (38.9%)	
III	271	188 (16.3%)	83 (39.3%)	
Missing	235	194 (16.8%)	41 (19.4%)	
Treatment				0.183
Non‐NAIT	621	516 (44.8%)	105 (49.8%)	
NAIT	742	636 (55.2%)	106 (50.2%)	
VRD	418	373 (58.6%)	45 (42.5%)	
VTD	170	140 (22.0%)	30 (28.3%)	
VPD	13	11 (1.7%)	2 (1.9%)	
IRD/ITD	33	27 (4.2%)	6 (5.7%)	
KRD/KPD	13	11 (1.7%)	2 (1.9%)	
DVD/DKD/DID	53	40 (6.3%)	13 (12.3%)	
DRD/DPD	15	13 (2.0%)	2 (1.9%)	
DVRD/DKRD	27	21 (3.3%)	6 (5.7%)	
ASCT				0.033
No	1032	860 (74.7%)	172 (81.5%)	
Yes	331	292 (25.3%)	39 (18.5%)	
Response				< 0.001
< PR	122	95 (8.2%)	27 (12.8%)	
PR	263	210 (18.2%)	53 (25.1%)	
VGPR	410	353 (30.6%)	57 (27.0%)	
CR and sCR	480	430 (37.3%)	50 (23.7%)	
Missing	88	64 (5.6%)	24 (11.4%)	

*Note:* BMPCs, bone marrow plasma cells; Hb, hemoglobin; DS, Durie–Salmon staging system; β2‐MG, β2 microglobulin; ISS, international staging system; LDH, lactate dehydrogenase; HRCA: high risk cytogenetic aberration; R‐ISS, revised international staging system; NAIT: novel agents‐based induction therapy; VRD, bortezomib, lenalidomide and dexamethasone; VTD, bortezomib, thalidomide and dexamethasone; VPD, bortezomib, pomalidomide and dexamethasone; IRD, ixazomib, lenalidomide and dexamethasone; ITD, ixazomib, thalidomide and dexamethasone; KRD, carfilzomib, lenalidomide and dexamethasone; DVD, daratumumab, bortezomib and dexamethasone; DKD, daratumumab, carfilzomib and dexamethasone; DID, daratumumab, ixazomib and dexamethasone; DRD, daratumumab, lenalidomide and dexamethasone; DPD, daratumumab, pomalidomide and dexamethasone; DVRD, daratumumab, bortezomib, lenalidomide and dexamethasone; DKRD, daratumumab, carfilzomib, lenalidomide and dexamethasone; ASCT, autologous stem cell transplantation; PR, partial response; VGPR, very good partial response; CR, complete response; sCR, stringent complete response.

^a^ LDH > 230 U/L in Qilu Hospital of Shandong University and Shandong Provincial Hospital Affiliated to Shandong First Medical University, LDH > 250 U/L in Fujian Medical University Union Hospital.

^b^ HRCA: del (17p), t (4;14), t (14;16), t (14;20), p53 mutation and 1q21 gain/amplification.

### 3.2 Responses to Induction Therapy

**FIGURE 2:**
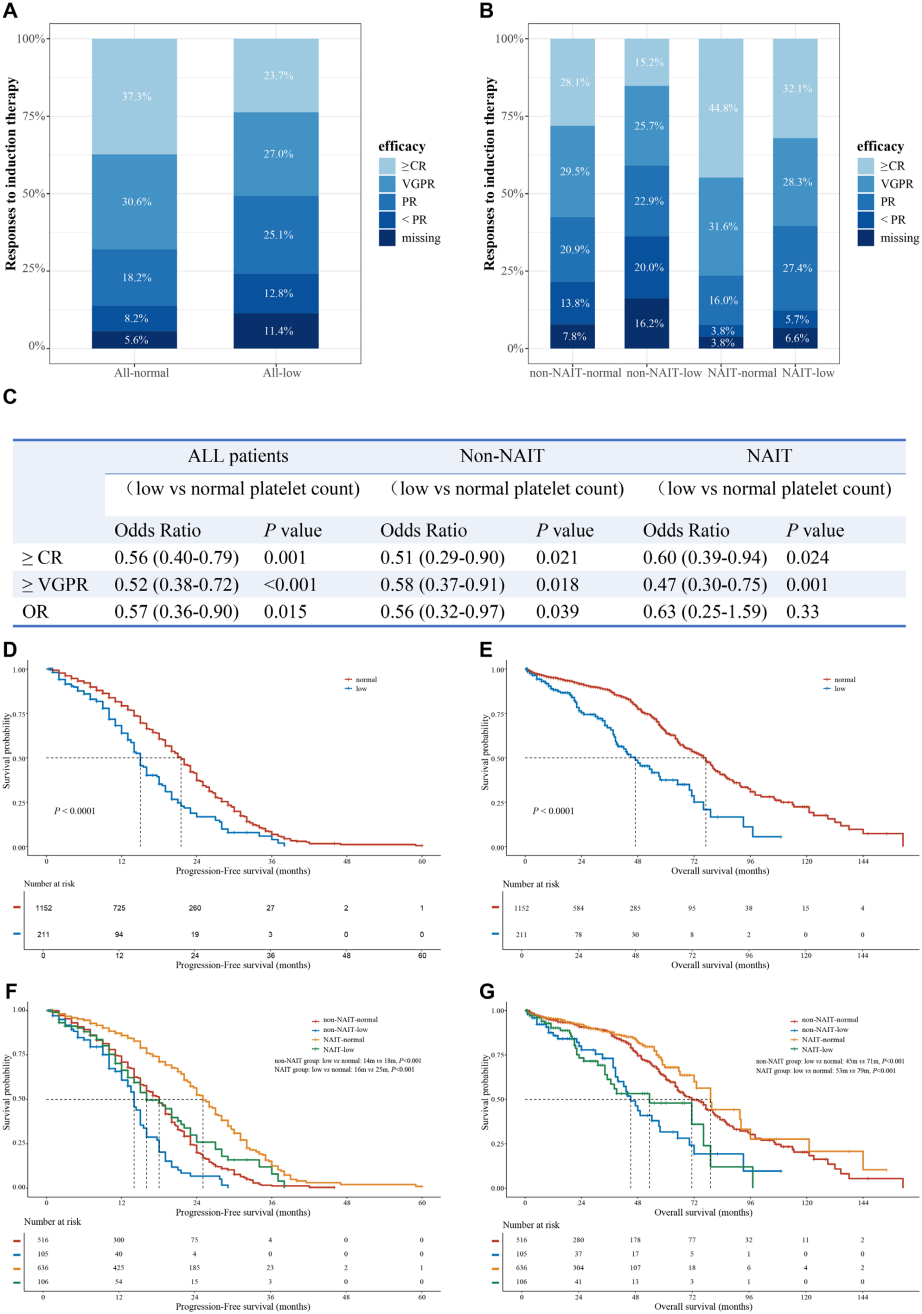
Responses and survival of NDMM patients with and without thrombocytopenia. (A) Responses to induction therapy among patients with normal and low platelet counts. (B) Responses of patients with and without thrombocytopenia receiving non‐NAIT and NAIT regimens, respectively. (C) Univariate logistic analysis of the effect of platelet count on efficacy in all patients, patients receiving non‐NAIT and NAIT regimens, respectively. (D) Progression‐free survival (PFS) between NDMM patients with normal and low platelet counts. (E) Overall survival (OS) between NDMM patients with normal and low platelet counts. (F) Progression‐free survival (PFS) of NDMM patients with normal and low platelet counts receiving non‐NAIT and NAIT regimens, respectively. (G) Overall survival (OS) of NDMM patients with normal and low platelet counts receiving non‐NAIT and NAIT regimens, respectively. NDMM, newly diagnosed multiple myeloma; NAIT, novel agents‐based induction therapy; CR, complete response; VGPR, very good partial response; PR, partial response; OR, overall response.

The overall response rates (ORR) were 86.1% and 75.8% in NDMM patients with normal and low platelet counts, respectively (*p* = 0.015, Figure 2A,C). NDMM patients with normal platelet counts achieved significantly better deep response (≥ VGPR) than thrombocytopenic patients (67.9% vs. 50.7%, *p* < 0.001, Figure 2A,C). The rate of sCR plus CR (≥ CR) in patients with normal platelet counts was also significantly higher than that in patients with thrombocytopenia (37.3% vs 23.7%, *p* = 0.001, Figure 2A,C). These results suggest that NDMM patients with thrombocytopenia have significantly worse efficacy.

NAIT regimens induced remarkably superior efficacy than non‐NAIT therapies in NDMM patients with normal and low platelet counts (Figure S1 and Table S1). To evaluate whether NAIT regimens showed benefits in NDMM patients with thrombocytopenia, we next analyzed the responses to NAIT in NDMM patients and found that the deep response (≥ VGPR) rate in patients with thrombocytopenia was significantly lower than that in patients with normal platelet counts (60.4% vs 76.4%, *p* = 0.001, Figure 2B,C), however, no significant difference was observed in OR rates between patients with or without thrombocytopenia (87.8% vs 92.4%, *p* = 0.33, Figure 2B,C). This indicates that NAIT can improve overall response but not deep response in NDMM patients with thrombocytopenia.

In NDMM patients receiving non‐NAIT regimens, both overall and deep response rates in patients with thrombocytopenia were significantly lower than those in patients without thrombocytopenia (ORR: 63.8% vs 78.5%, *p* = 0.039; ≥ VGPR: 40.9% vs 57.6%, *p* = 0.018; respectively, Figure 2B,C).

### 3.3 Survival Outcomes

With a median follow‐up of 27 months, both PFS and OS in NDMM patients with thrombocytopenia were significantly worse than those in patients with normal platelet counts (median PFS: 15 vs 21.5 months, *p* < 0.001; median OS: 47 vs 77 months, *p* < 0.001, respectively; Figure 2D,E). In subgroup analyses of survival, PFS, and OS of patients in different DS, ISS, and R‐ISS stages were compared between patients with and without thrombocytopenia (Figure S2). In general, the outcomes in patients with thrombocytopenia at different stages were inferior to those in patients with normal platelet counts.

Induction regimens and platelet counts were included in Kaplan–Meier analysis to further investigate the impacts of these two factors on PFS and OS. As shown in Figure 2F,G, among patients with NAIT regimens, both PFS and OS of patients with thrombocytopenia were significantly shorter than those of patients with normal platelet counts (median PFS: 16 vs 25 months, *p* < 0.001, median OS: 53 vs 79 months, *p* < 0.001, respectively). In patients with non‐NAIT regimens, the outcomes of patients with thrombocytopenia were also significantly worse than those of patients with normal platelet counts (median PFS: 14 vs 18 months, *p* < 0.001; median OS: 45 vs 71 months, *p* < 0.001, respectively). Neither NAIT nor non‐NAIT regimens improved the survival of patients with thrombocytopenia.

Univariate and multivariate Cox analyses for PFS and OS were presented in Tables 2 and S2, respectively. In the multivariate analysis for PFS (Table 2), thrombocytopenia [hazard ratio (95% confidence interval, 95% CI) 1.40 (1.14–1.72), *p* = 0.001] and R‐ISS III [1.60 (1.13–2.28), *p* = 0.009] were associated with worse PFS. In contrast, NAIT [0.52 (0.45–0.60), *p* < 0.001], ASCT [0.71 (0.60–0.85), *p* < 0.001], and achieving deep response (≥ VGPR) [0.53 (0.45–0.62), *p* < 0.001] were associated with better PFS. Similarly, in the multivariate analysis for OS (Table S2), thrombocytopenia [1.74 (1.29–2.35), *p* < 0.001] and R‐ISS stage III [2.07 (1.10–3.90), *p* = 0.023] were associated with worse OS. In contrast, ASCT [0.69 (0.49–0.97), *p* = 0.033] and achieving deep response (≥ VGPR) [0.57 (0.45–0.73), *p* < 0.001] were associated with better OS.

**TABLE 2:** Univariate and multivariate Cox analyses for PFS.

Variables	Univariate analysis		Multivariate analysis	
	Hazard ratio (95% CI)	*p*	Hazard ratio (95% CI)	*p*
Male	1.01 (0.88–1.16)	0.867	—	—
Age > 60 years	0.95 (0.83–1.09)	0.488	—	—
Thrombocytopenia	1.76 (1.46–2.13)	**< 0.001**	1.40 (1.14–1.72)	**0.001**
Ca ≥ 2.65 mmol/L	1.34 (1.09–1.65)	**0.006**	1.20 (0.96–1.50)	0.107
Cr ≥ 177umol/L	1.40 (1.17–1.67)	**< 0.001**	0.99 (0.81–1.22)	0.94
Hb < 85 g/L	1.46 (1.27–1.69)	**< 0.001**	1.18 (1.00–1.39)	0.055
Bone destruction < 3 sites	reference			
Bone destruction ≥ 3 sites	1.10 (0.95–1.27)	0.19	—	—
Missing	1.26 (0.96–1.65)	0.09		
ISS I	reference			
ISS II	1.10 (0.91–1.32)	0.326	1.04 (0.80–1.35)	0.764
ISS III	1.60 (1.35–1.90)	**< 0.001**	1.01 (0.77–1.31)	0.953
R‐ISS I	reference			
R‐ISS II	1.37 (1.09–1.74)	**0.008**	1.13 (0.83–1.55)	0.431
R‐ISS III	2.38 (1.84–3.09)	**< 0.001**	1.60 (1.13–2.28)	**0.009**
Missing	1.59 (1.22–2.08)	0.001	1.13 (0.84–1.52)	0.427
NAIT	0.47 (0.41–0.54)	**< 0.001**	0.52 (0.45–0.60)	**< 0.001**
ASCT	0.59 (0.50–0.70)	**< 0.001**	0.71 (0.60–0.85)	**< 0.001**
< VGPR	Reference			
≥ VGPR	0.44 (0.38–0.52)	**< 0.001**	0.53 (0.45–0.62)	**< 0.001**
Missing	0.82 (0.59–1.13)	0.215	0.83 (0.60–1.14)	0.244

*Note:* 95% CI, 95% confidence interval. Values in bold denote statistical significance at *p* < 0.05.

Abbreviations: ASCT, autologous stem cell transplantation; Hb, hemoglobin; ISS, international staging system; NAIT, novel agents‐based induction therapy; PFS, progression‐free survival; R‐ISS, revised international staging system; VGPR, very good partial response.

### 3.4 Nomogram Construction and Validation

**FIGURE 3:**
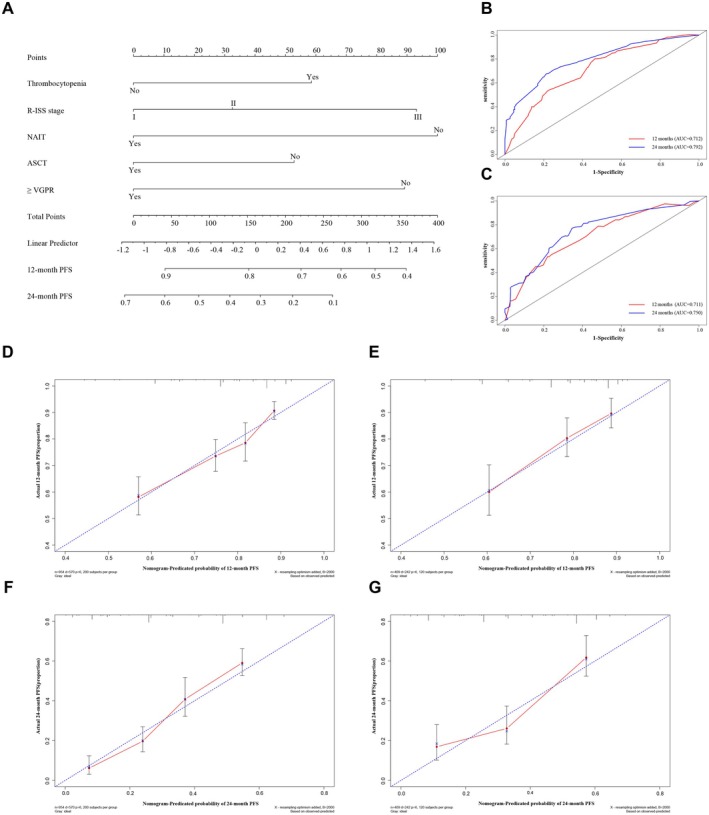
The nomogram for the prediction of PFS in NDMM patients. (A) Thrombocytopenia, R‐ISS stage, NAIT, ASCT, and deep response (≥ VGPR) were used to construct a nomogram predicting 12 and 24‐month PFS. (B) The area under the curve (AUC) for the training nomogram model demonstrated values of 0.712 at 12 months and 0.792 at 24 months. (C) The validation nomogram model yielded AUC of 0.711 at 12 months and 0.750 at 24 months. (D‐G) Calibration curves of the nomogram in terms of the agreement between predicted and observed 12‐month PFS in the training group (D) and validation group (E), and 24‐month PFS in the training group (F) and in the validation group (G), respectively. PFS, progression‐free survival; NDMM, newly diagnosed multiple myeloma; R‐ISS, Revised International Staging System; NAIT, novel agents‐based induction therapy; ASCT, autologous stem cell transplantation; VGPR, very good partial response.

Based on the results of multivariate regression analysis, thrombocytopenia, R‐ISS stage, NAIT, ASCT, and deep response (≥ VGPR) were used to construct a nomogram predicting 12 and 24‐month PFS in the complete dataset (Figure 3A). The baseline characteristics of the training group and validation group were presented in Table S3 with no significant bias. ROC analysis was used to assess the discrimination of the nomogram. The area under the curve (AUC) for the training nomogram model demonstrated values of 0.712 at 12 months and 0.792 at 24 months, whereas the validation nomogram model yielded AUCs of 0.711 and 0.750 at 12 and 24‐month intervals, respectively (Figure 3B,C). The calibration curves for the probability of 12 and 24‐month PFS demonstrated a good agreement between the actual reported and the predicted PFS (Figure 3D–G). Our nomogram model effectively predicted PFS for NDMM patients.

## 4 Discussion

In this multicenter retrospective study, we analyzed the clinical characteristics, responses to induction therapy, and survival of NDMM patients with and without thrombocytopenia, performed regression analysis, and constructed a nomogram model to predict PFS. We found that NDMM patients with thrombocytopenia showed a larger number of bone marrow plasma cells, more advanced disease stages, and worse outcomes than patients without thrombocytopenia. NAIT could improve overall response, but not deep response or survival in NDMM patients with thrombocytopenia. Multivariate regression analysis proposed that thrombocytopenia, together with R‐ISS stage III, NAIT, ASCT, and deep response, were significantly correlated with survival.

Thrombocytopenia is less common in NDMM patients, and its cut‐off value varies across different studies. This study, conducted in China, defined thrombocytopenia as a platelet count of less than 100,000/uL. In this situation, the incidence of thrombocytopenia in NDMM patients was approximately 15%, which was in line with previously reported data [7, 8, 10]. The presence of thrombocytopenia in NDMM patients significantly correlated with invasive clinical manifestations, including high myeloma burden, severe anemia, low albumin levels, renal failure, and elevated β2‐MG and LDH levels. These factors, along with HRCA, are important indicators for MM disease staging and risk stratification. Charalampous et al. analyzed the association of thrombocytopenia with disease burden, HRCA, and survival in NDMM patients from Mayo Clinic and found that thrombocytopenia was associated with mortality independently [10]. They demonstrated that thrombocytopenia was significantly associated with t (4;14) and t (14;16) [10]. Due to the cost and accessibility of the fluorescence in situ hybridization (FISH) test, part of the NDMM patients in our study failed to complete the FISH test, resulting in some missing data in the risk stratification of cytogenetics. We failed to present the exact correlation between thrombocytopenia and a specific cytogenetic abnormality. However, we found that NDMM patients with thrombocytopenia had a higher proportion of HRCA based on the available data. Compared to NDMM patients with normal platelet counts, those with thrombocytopenia were more frequently classified into DS stage III, ISS stage III, and R‐ISS stage III. Moreover, thrombocytopenic patients in stage III demonstrated the worst OS and PFS. These findings are consistent with previous studies indicating that thrombocytopenic patients carry a higher tumor burden, more aggressive disease, and an overall poor prognosis [9, 10]. Platelets, which are components of blood, are differentiated and matured from hematopoietic stem cells [27]. The number of platelets reflects the hematopoietic function and maturation status of the bone marrow [28]. Meanwhile, plasma cell malignant proliferation and cytokine secretion, which form a negative microenvironment [29, 30], can decrease platelet production [9].

Interestingly, our study demonstrated that male NDMM patients were more likely to have thrombocytopenia. Other studies reported similar results that the percentage of male patients with thrombocytopenia was higher than that of female myeloma patients [9, 10], suggesting that male patients were susceptible to thrombocytopenia at diagnosis. This observation may be attributed to the potential role of androgens in upregulating thrombopoietin (TPO) synthesis [31]. Notably, multiple studies have demonstrated an inverse correlation between circulating TPO levels and platelet counts in multiple myeloma [32, 33]. NDMM patients with or without thrombocytopenia in our study received comparable induction regimens. However, the percentage of NDMM patients with normal platelet count receiving ASCT was a little higher than that of patients with thrombocytopenia, mainly due to the better performance status and younger age in patients with normal platelet count.

Survival in MM patients has improved significantly during the past two decades in China and around the world [34, 35, 36, 37]. Numerous combinations for initial therapy have been developed based on novel agents that have shown apparent efficacy [12, 38]. Recent studies have established and further consolidated triplet and quadruplet regimens in the management of MM patients [39, 40, 41, 42]. The most commonly recommended induction regimens are triplet and quadruplet regimens consisting of proteasome inhibitors, immunomodulatory agents, and monoclonal antibodies [3, 37]. To investigate whether NAIT can overcome the poor prognosis associated with thrombocytopenia, we compared the outcomes and survival of NDMM patients with and without thrombocytopenia receiving NAIT regimens. We found that NDMM patients with thrombocytopenia had poor outcomes and survival. NAIT significantly improved the ORR of patients with thrombocytopenia close to that of patients with a normal platelet count. But unfortunately, NAIT failed to achieve a satisfactory deep response and thus did not prolong their survival in NDMM patients with thrombocytopenia. NDMM patients with a normal platelet count who received NAIT had the longest PFS and OS, followed by those with a normal platelet count receiving non‐NAIT and those with thrombocytopenia receiving NAIT, respectively. NDMM patients with thrombocytopenia who received non‐NAIT had the worst survival. NAIT regimens induced better outcomes and improved survival of NDMM patients compared with non‐NAIT treatments. The improvement of the quality of response is associated with better disease control and longer survival [43, 44, 45]. The achievement of maximal response should be strongly considered in eligible patients.

The prognosis evaluation and risk stratification of MM patients were complex and variable [46, 47, 48, 49, 50]. We performed univariate and multivariate analyses for PFS and OS, and found that thrombocytopenia, R‐ISS stage III, NAIT, ASCT, and deep response were significantly correlated with survival. These five factors were used to construct a nomogram to predict 12 and 24‐month PFS with reliable predictive ability. Recently, Maura F et al. integrated clinical, genomic, and therapeutic data to build a model predicting individualized risk in NDMM patients [51]. This model is an online available tool including patients' demographics, ISS, IGH translocations, genomics, induction, and post‐induction therapies. They developed an individualized risk‐prediction model enabling personally tailored therapeutic decisions for NDMM patients. In our study, induction therapy, response to induction therapy, and ASCT were also included in the construction of the nomogram model based on the results of multivariate Cox regression analysis, and these factors played important roles in predicting PFS. Most existing risk stratification models in multiple myeloma have not included platelet count as a laboratory feature [37, 52], unless the MPSS risk model, which incorporates platelet count and improves the risk estimation in NDMM patients [8]. The inclusion of thrombocytopenia as a high‐risk factor in the prognosis of multiple myeloma is controversial. Our results suggest that NDMM patients with thrombocytopenia have poor prognosis, similar to that of patients with high‐risk MM [47, 52].

Our findings are based on a retrospective observational study, which has certain limitations. First, the most commonly used two induction therapies in our study are VRD and VTD. The percentage of patients receiving induction regimens consisting of daratumumab and carfilzomib is relatively low. Therefore, the effects of regimens composed of monoclonal antibodies, new generation proteasome inhibitors, and immunomodulatory agents in NDMM patients with thrombocytopenia need to be further confirmed. Second, we used random forest interpolation in constructing the nomogram and dummy variables in multifactor Cox regression to deal with some missing data. Although good internal verification results are obtained, this model needs to be further validated by external data.

In conclusion, thrombocytopenia in NDMM patients significantly affects responses to induction therapy and survival. Thrombocytopenia should be regarded as an independent prognostic factor in the risk stratification of Chinese NDMM patients.

## Author Contributions

All data are available from the corresponding author upon reasonable request. CRediT authorship contribution statement: Hai Zhou: conceptualization, methodology, data curation, funding acquisition, project administration, writing, original draft, writing, review and editing, supervision, and resources. Ping Chen: conceptualization, methodology, data curation, funding acquisition, project administration, writing, original draft, writing, review and editing, supervision, and resources. Xiaojing Li: methodology, software, data curation, investigation, validation, formal analysis, writing, original draft, writing, review and editing. Xiaohui Lai: data curation, investigation, validation, and writing, review and editing. Xiaolin Wang: data curation, investigation, validation, and writing, review and editing. Qiang Liu: validation, and writing, review and editing. Xin Liu: validation, and writing, review and editing. Luqun Wang: validation, and writing, review and editing. Jun Peng: validation, funding acquisition, and writing, review and editing.

## Conflicts of Interest

The authors declare no conflicts of interest.

## Supporting information


**Figure S1.** Responses of NDMM patients receiving non‐NAIT and NAIT regimens.
**Figure S2**. Progression‐free survival (PFS) and overall survival (OS) of NDMM patients in different DS (A and B), ISS (C and D), and R‐ISS (E and F) stages were compared between patients with and without thrombocytopenia.
**Table S1.** Univariate logistic analysis of the effect of induction therapies on efficacy in all patients, patients with normal and low platelet count, respectively.
**Table S2.** Univariate and multivariate Cox analyses for overall survival (OS).
**Table S3.** Baseline characteristics for the entire cohort, training group, and validation group after random forest interpolation.

## Data Availability

The data that support the findings of this study are available from the corresponding author upon reasonable request.
