# Supplementary material for: Prognostic Implications of Thrombocytopenia in Chinese Patients With Newly Diagnosed Multiple Myeloma
Source: Cancer Med. 2025 Nov 2;14(21):e71353. doi: 10.1002/cam4.71353 (PMC12579819; doi:10.1002/cam4.71353)
Supplement: Supplementary file 1 — Figure S1. Responses of NDMM patients receiving non‐NAIT and NAIT regimens. Figure S2. Progression‐free survival (PFS) and overall survival (OS) of NDMM patients in different DS (A and B), ISS (C and D), and R‐ISS (E and F) stages were compared between patients with and without thrombocytopenia. Table S1. Univariate logistic analysis of the effect of induction therapies on efficacy in all patients, patients with normal and low platelet count, respectively. Table S2. Univariate and multivariate Cox analyses for overall survival (OS). Table S3. Baseline characteristics for the entire cohort, training group, and validation group after random forest interpolation. [file CAM4-14-e71353-s001.docx]

Prognostic implications of thrombocytopenia in Chinese patients with newly diagnosed multiple myeloma

Supplementary Figures and Tables


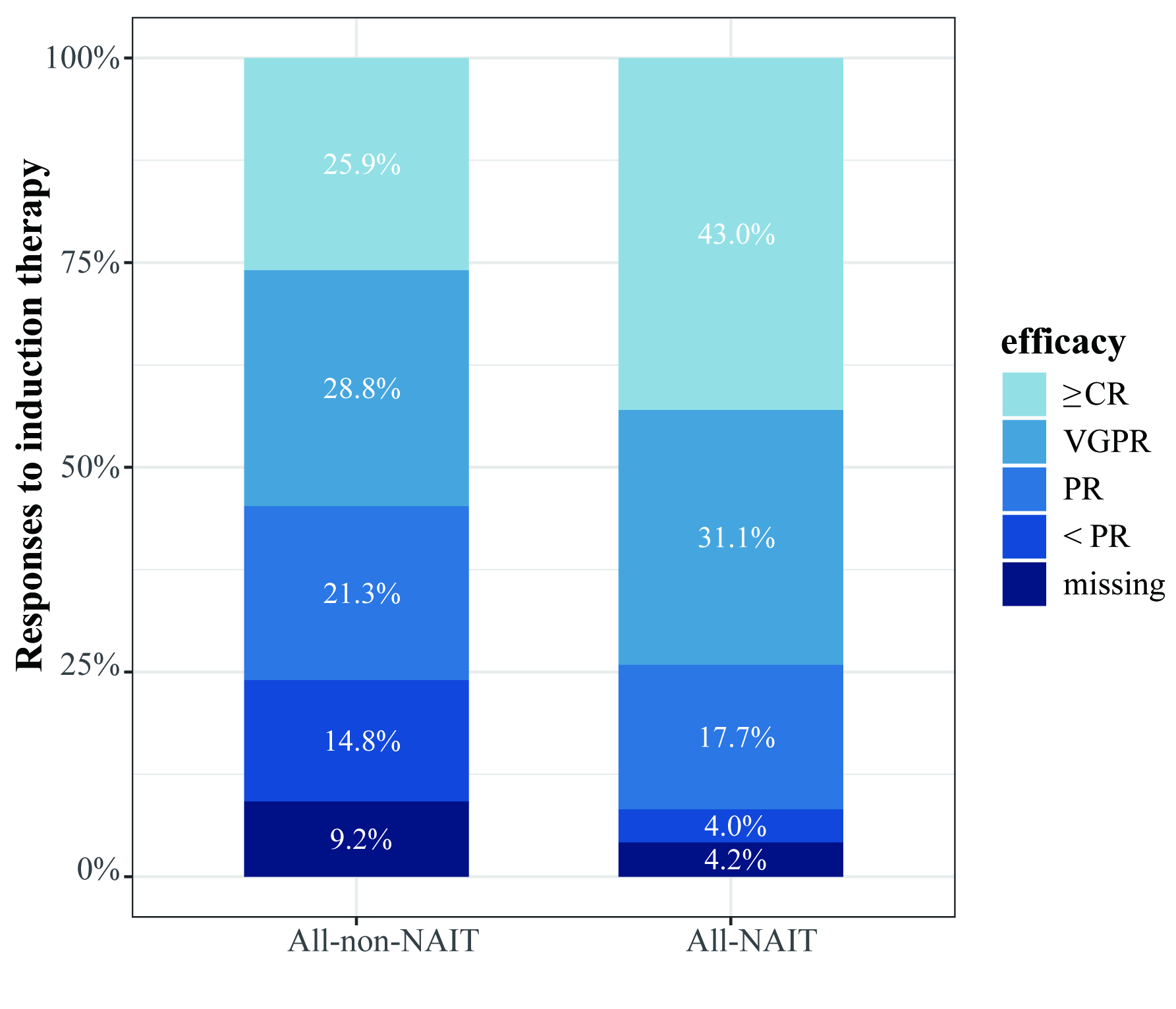


Figure S1. Responses of NDMM patients receiving non-NAIT and NAIT regimens.

Abbreviations: NDMM, newly diagnosed multiple myeloma; NAIT, novel agents-based induction therapy; All-non-NAIT, patients receiving non-NAIT regimens; All-NAIT, patients receiving NAIT regimens.


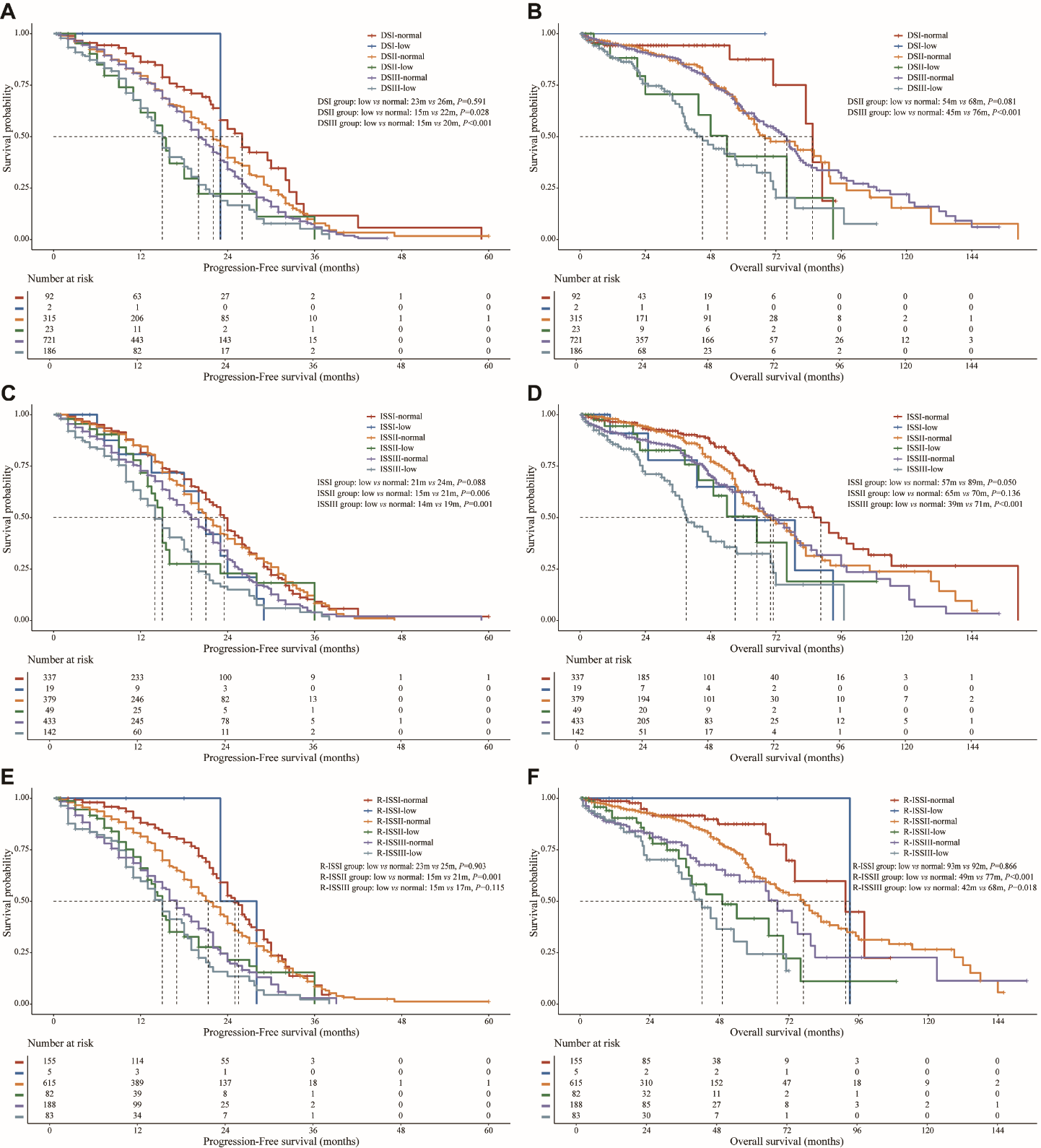
Figure S2. Progression free survival (PFS) and overall survival (OS) of NDMM patients in different DS (A and B), ISS (C and D) and R-ISS (E and F) stages were compared between patients with and without thrombocytopenia.

NDMM, newly diagnosed multiple myeloma; normal, normal platelet count; low, low platelet count; DS, Durie-Salmon stage; ISS, International Staging System; R-ISS, Revised International Staging System.

Table S1: Univariate logistic analysis of the effect of induction therapies on efficacy in all patients, patients with normal and low platelet count, respectively.

|  | ALL patients  （NAIT *vs* non-NAIT) | | Patients with normal platelet count  （NAIT *vs* non-NAIT ) | | Patients with low platelet count  （NAIT *vs* non-NAIT) | |
| --- | --- | --- | --- | --- | --- | --- |
|  | Odds Ratio (95% CI) | *P* value | Odds Ratio (95% CI) | *P* value | Odds Ratio (95% CI) | *P* value |
| ≥ CR | 2.04 (1.61-2.58) | <0.001 | 1.99 (1.55-2.56) | <0.001 | 2.35 (1.19-4.66) | 0.014 |
| ≥ VGPR | 2.25 (1.76-2.87) | <0.001 | 2.33 (1.78-3.05) | <0.001 | 1.91 (1.06-3.44) | 0.030 |
| OR | 4.43 (2.88-6.79) | <0.001 | 4.30 (2.66-6.94) | <0.001 | 4.86 (1.86-12.69) | 0.001 |

95% CI, 95% confidence interval; NAIT, novel agents-based induction therapy. CR, complete response; VGPR, very good partial response; OR, overall response.

Table S2: Univariate and multivariate Cox analyses for overall survival (OS).

|  | Univariate analysis | | Multivariate analysis | |
| --- | --- | --- | --- | --- |
| Variables | Hazard Ratio (95% CI) | *P* value | Hazard Ratio (95% CI) | *P* value |
| Male | 1.28 (1.02-1.60) | 0.030 | 1.21 (0.97-1.53) | 0.098 |
| Age >60 year | 1.30 (1.04-1.62) | 0.020 | 1.15 (0.91-1.46) | 0.238 |
| Thrombocytopenia | 2.41 (1.83-3.16) | < 0.001 | 1.74 (1.29-2.35) | < 0.001 |
| Ca >2.65mmol/L | 1.21 (0.84-1.72) | 0.307 | - | - |
| Cr >177umol/L | 1.53 (1.16-2.01) | 0.002 | 1.09 (0.80-1.50) | 0.578 |
| Hb <85g/L | 1.60 (1.28-2.00) | < 0.001 | 1.08 (0.83-1.40) | 0.574 |
| Bone destruction > 3 sites | 0.98 (0.77-1.23) | 0.847 | - | - |
| ISS I | reference |  |  |  |
| ISS II | 1.43 (1.04-1.95) | 0.027 | 1.34 (0.88-2.04) | 0.179 |
| ISS III | 2.20 (1.65-2.94) | < 0.001 | 1.27 (0.84-1.91) | 0.255 |
| R-ISS I | reference |  |  |  |
| R-ISS II | 1.87 (1.14-3.05) | 0.013 | 1.15 (0.63-2.08) | 0.650 |
| R-ISS III | 3.70 (2.21-6.18) | < 0.001 | 2.07 (1.10-3.90) | 0.023 |
| missing | 2.09 (1.25-3.5) | 0.005 | 1.38 (0.78-2.42) | 0.269 |
| NAIT | 0.84 (0.66-1.06) | 0.136 | - | - |
| ASCT | 0.53 (0.39-0.73) | < 0.001 | 0.69 (0.49-0.97) | 0.033 |
| < VGPR | reference |  |  |  |
| ≥ VGPR | 0.51 (0.40-0.64) | < 0.001 | 0.57 (0.45-0.73) | < 0.001 |
| missing | 0.82 (0.52-1.30) | 0.41 | 0.99 (0.62-1.58) | 0.967 |

95%CI, 95% confidence interval; Hb, hemoglobin; ISS, international staging system; R-ISS, revised international staging system; NAIT: novel agents-based induction therapy; ASCT, autologous stem cell transplantation; VGPR, very good partial response.

Table S3: Baseline characteristics for the entire cohort, training group and validation group after random forest interpolation.

|  | Entire cohort (N=1363) | Training group (N=954) | Validation group (N=409) |
| --- | --- | --- | --- |
| Male, n (%) | 753 (55.25%) | 532 (55.77%) | 221 (54.03%) |
| Age (years) | 60 (27-88) | 60 (29-88) | 61 (27-86) |
| Thrombocytopenia, n (%) | 211 (15.48%) | 151 (15.83%) | 60 (14.67%) |
| Ca ≥ 2.65mmol/L, n (%) | 163 (11.96%) | 103 (10. 80%) | 60 (14.67%) |
| Cr ≥177umol/L, n (%) | 238 (17.46%) | 169 (17.71%) | 69 (16.87%) |
| Hb <85g/L, n (%) | 506 (37.12%) | 357 (37.42%) | 149 (36.43%) |
| Bone destruction ≥ 3 sites, n (%) | 602 (44.17%) | 433 (45.39%) | 169 (41.32%) |
| DS stage, n (%) |  |  |  |
| I | 95 (6.97%) | 64 (6.70%) | 31 (7.58%) |
| II | 361 (26.49%) | 245 (25.68%) | 116 (28.36%) |
| III | 907 (66.54%) | 645 (67.61%) | 262 (64.06%) |
| β2-MG ≥ 5.5 mg/L, n (%) | 579 (42.48%) | 401 (42.03%) | 178 (43.52%) |
| Albumin < 35 g/L, n (%) | 559 (41.01%) | 388 (40.67%) | 171 (41.81%) |
| ISS stage, n (%) |  |  |  |
| I | 356 (26.12%) | 249 (26.10%) | 107 (26.16%) |
| II | 428 (31.40%) | 304 (31.87%) | 124 (30.32%) |
| III | 579 (42.48%) | 401 (42.03%) | 178 (43.52%) |
| Elevated LDH (U/L), n (%) | 316 (23.18%) | 217 (22.75%) | 99 (24.21%) |
| HRCA, n (%) | 952 (69.85%) | 664 (69.60%) | 288 (70.42%) |
| R-ISS stage, n (%) |  |  |  |
| I | 160 (11.74%) | 115 (12.05%) | 45 (11.00%) |
| II | 931 (68.31%) | 656 (68.76%) | 275 (67.24%) |
| III | 272 (19.96%) | 183 (19.18%) | 89 (21.76%) |
| NAIT, n (%) | 742 (54.44%) | 519 (54.40%) | 223 (54.52%) |
| ASCT, n (%) | 331 (24.28%) | 245 (25.68%) | 86 (21.03%) |
| ≥ VGPR, n (%) | 978 (71.25%) | 693 (72.64%) | 285 (69.68%) |
| Median OS (months) | 72 | 71 | 77 |
| Median PFS (months) | 20 | 20 | 20 |

Hb, hemoglobin; DS, Durie-Salmon staging system; β2-MG, β2 microglobulin; ISS, international staging system; LDH, lactate dehydrogenase; HRCA: high risk cytogenetic aberration; R-ISS, revised international staging system; NAIT: novel agents-based induction therapy; ASCT, autologous stem cell transplantation; VGPR, very good partial response; OS, overall survival; PFS, progression free survival.
